# Douyin and Bilibili as sources of information on lung cancer in China through assessment and analysis of the content and quality

**DOI:** 10.1038/s41598-024-70640-y

**Published:** 2024-09-04

**Authors:** Fanyi Zeng, Weilin Zhang, Menghui Wang, Hejin Zhang, Xiaoyi Zhu, Hui Hu

**Affiliations:** 1 Department of Health Management Center, The Second Affiliated Hospital of Nanchang University, Jiangxi Medical College, Nanchang University, Nanchang, 330006 Jiangxi Province China; 2 Huan Kui Academy, Jiangxi Medical College, Nanchang University, Nanchang, 330006 Jiangxi Province China; 3 The Second Clinical Medical College of Nanchang University, Jiangxi Medical College, Nanchang University, Nanchang, 330006 Jiangxi Province China

**Keywords:** Lung cancer, Social media, Short videos, Information quality, Douyin, Bilibili, Cancer, Lung cancer

## Abstract

Lung cancer has emerged as a major global public health concern. With growing public interest in lung cancer, online searches for related information have surged. However, a comprehensive evaluation of the credibility, quality, and value of lung cancer-related videos on digital media platforms remains unexamined. This study aimed to assess the informational quality and content of lung cancer-related videos on Douyin and Bilibili. A total of 200 lung cancer-related videos that met the criteria were selected from Douyin and Bilibili for evaluation and analysis. The first step involved recording and analyzing the basic information provided in the videos. Subsequently, the source and type of content for each video were identified. All videos’ educational content and quality were then evaluated using JAMA, GQS, and Modified DISCERN. Douyin videos were found to be more popular in terms of likes, comments, favorites, and shares, whereas Bilibili videos were longer in duration (*P* < .001). The majority of video content on both platforms comprised lung cancer introductions (31/100, 31%), with medical professionals being the primary source of uploaded videos (Douyin, n = 55, 55%; Bilibili, n = 43, 43%). General users on Douyin scored the lowest on the JAMA scale, whereas for-profit businesses scored the highest (2.50 points). The results indicated that the videos’ informational quality was insufficient. Videos from science communications and health professionals were deemed more reliable regarding completeness and content quality compared to videos from other sources. The public should exercise caution and consider the scientific validity when seeking healthcare information on short video platforms.

## Introduction

Lung cancer is the leading cause of cancer-related deaths globally, particularly in China^1^. In 2018, China accounted for approximately 37.56% of global lung cancer mortality rates^2^.

Lung cancer is a malignant tumor originating from abnormal cell growth in the lung tissue. Smoking is widely recognized as the primary risk factor for lung cancer^3^. Environmental pollution and genetic factors also contribute to the development of lung cancer. Certain gene mutations are more prevalent in smokers^4^. Individual and regional variations in the presentation and characteristics of lung cancer are important to acknowledge.

Social media platforms have become increasingly popular among patients as a means of seeking information and establishing connections. Evaluating the effectiveness of these platforms in disseminating information about lung cancer is crucial.

Effective health communication is paramount for enhancing disease prevention and management. Low-income, ethnically diverse populations often lack sufficient guidance in recognizing and managing lung cancer. Previous studies have highlighted the importance of decision aids and the understanding of lung cancer screening processes^5,6^. Social media, particularly video formats, plays a significant role in health communication and interventions. Platforms such as Bilibili and Douyin have increased the distribution of health information in an engaging and user-friendly manner. However, the quality of lung cancer-related videos on these platforms has not been comprehensively evaluated^7^. To address this research gap, this study evaluated the top 100 lung cancer-related videos on Bilibili and Douyin using established assessment systems such as JAMA, GQS, and Modified DISCERN. These systems are designed to assess the credibility of videos related to diseases or treatments^8,9^. The research team conducted a comparative analysis of videos from different sources, evaluating both content quality and video reliability.

## Materials and methods

### Search strategy

To minimize bias in the analysis of newly uploaded videos, a search was conducted on September 12, 2023, on two video-sharing platforms, Bilibili and Douyin in China^10^. The keyword used for the search was “lung cancer”. To mitigate the impact of historical searches and intelligent recommendations, new accounts were created on each platform. The videos were reviewed in descending order of their rankings, as determined by the respective global ranking algorithms of the platforms. Simultaneously, detailed information, including user ID, title, and duration was collected for all analyzed videos to facilitate further analysis.

During this search, two investigators independently reviewed and evaluated the aforementioned videos. In cases of discrepancies or disagreements in their assessments, the authors were consulted to reach a unanimous conclusion.

### Video selection criteria

**Fig. 1:**
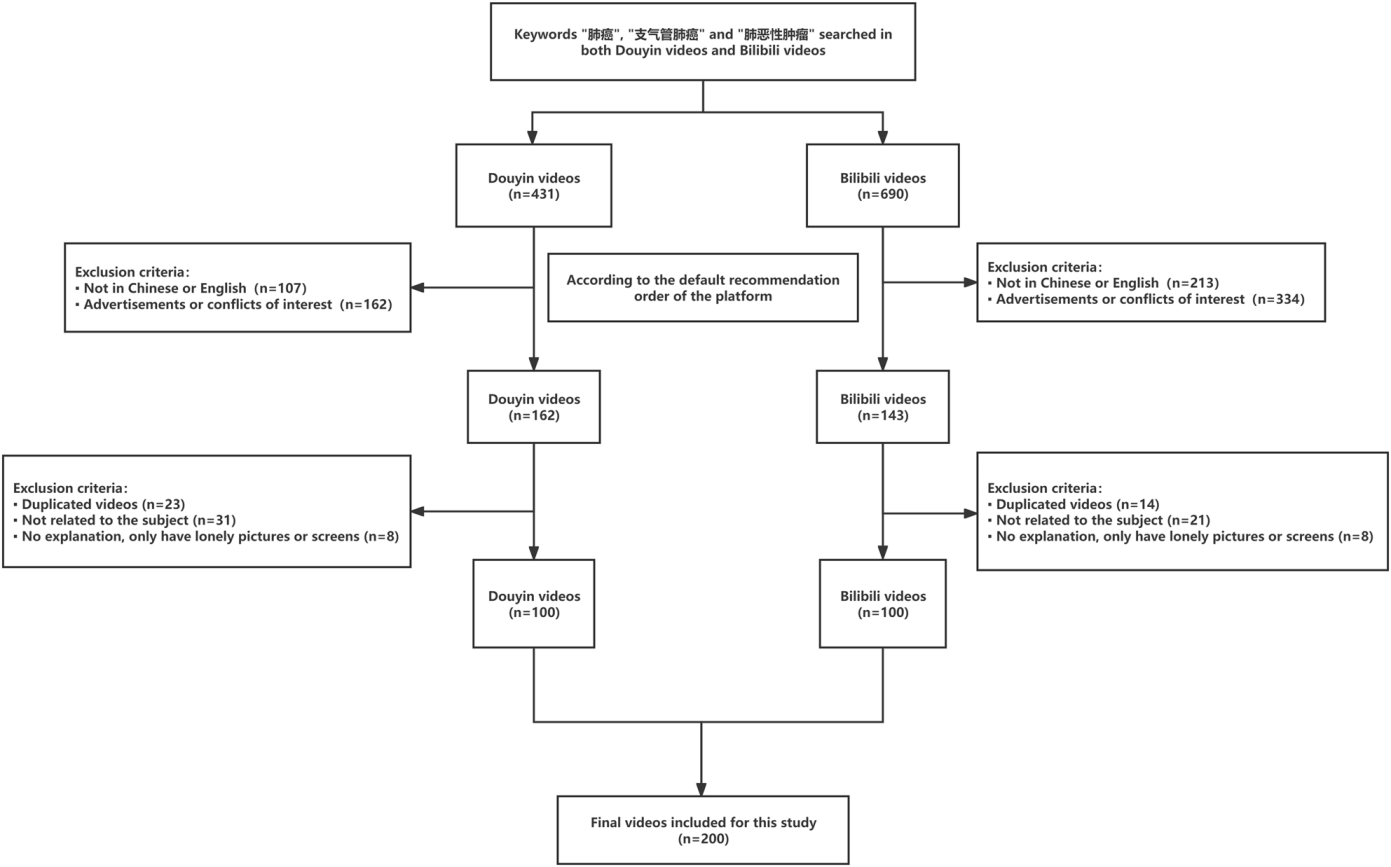
Search strategy and video screening procedure.

This study analyzed videos sourced from Bilibili and Douyin, which were listed based on the platforms’ default recommendation order. Exclusion criteria were as follows: videos in languages other than Chinese or English, advertisements or conflicts of interest, repetitive videos, pictorial content without accompanying explanations, and videos unrelated to the topic. After applying these exclusion criteria, the remaining 100 videos from each platform were used for further analysis^11^ (Fig. 1).

### Collection of video features

A total of 200 videos from Douyin and Bilibili were collected and analyzed in this study. Detailed information was gathered for each video, including the title, upload platform, upload time, source (health professionals, general users, science communications, news agencies, nonprofit organizations, and for-profit organizations), duration, views, likes, comments, and content type (early signals, terminal symptoms, etiologies and causations, scientific introductions, and treatment methods). Additionally, the JAMA, GQS, and Modified DISCERN scales were employed to evaluate the quality and value of the videos (Table S1).

The videos analyzed in this study were categorized into two main groups based on their source: individual users and organizational users. Individual users comprised health professionals, such as doctors and nurses, as well as general users and science communication contributors, like science popularization writers^12^. Conversely, organizational users consisted of news organizations, nonprofits, and for-profit organizations. Nonprofit organizations were defined as entities primarily focused on collective, public, or social interests, which could encompass public hospitals^13^. For-profit organizations were characterized as entities pursuing commercial interests^14^.

The video content analysis focused on five key aspects related to lung cancer: early signals, terminal symptoms, etiologies and causations, scientific introductions, and treatment methods.

### Assessment of each video

In this study, the content of videos was evaluated using three established standard scales: JAMA, GQS, and Modified DISCERN. These scales were employed to assess the quality and effectiveness of the analyzed videos.

Our comprehensive approach focused on evaluating the content, reliability, and informational quality of the videos. The reliability assessment utilized the JAMA criteria, which is based on four predetermined questions: authorship, signature, currency, and disclosure. Each criterion was assigned 1 point, resulting in a maximum possible score of 4 points. The assessment examined whether the video provided information about the author, included copyright information and references/sources, indicating the initial date and subsequent updates, and disclosed conflicts of interest, funding, sponsorship, advertising support, or video ownership^15^. The GQS was used to evaluate the dimensions of quality, flow, comprehensiveness, and usefulness on a five-point scale ranging from 1 (indicating poor quality) to 5 (indicating very good flow and quality). Higher scores indicated better quality^16^. To evaluate the information quality of the videos, the widely used Modified DISCERN tool was employed. Given that the included videos pertained to the medical field, a modified version of Modified DISCERN was used, focusing on five aspects: simplicity, relevance, traceability, robustness, and fairness. The Modified DISCERN consisted of five questions, with positive responses assigned a point and negative responses being assigned zero points^17^.

### Statistical analysis

In this study, data analysis was conducted using the Statistical Package for Social Sciences version 22 (IBM, Armonk, NY, USA). Descriptive statistics, including as mean, standard deviation, frequency, median, minimum, and maximum values, were calculated. Descriptive statistics are reported as median (minimum, maximum). The normality of the collected data was assessed using the Shapiro–Wilk test. For quantitative data following a normal distribution, values are expressed as mean ± standard deviation (x̄ ± SD). The nonparametric Kruskal–Wallis test was employed to identify significant differences between the independent variables of the two groups. In cases where the Kruskal–Wallis test indicated significant results, the Dunn-Bonferroni method was applied for pairwise comparisons. Spearman’s test was utilized to assess the correlation between independent variables. The results were considered statistically significant with a 95% confidence interval and a significance level of *P* < 0.05. The Bonferroni adjustment, automatically performed by IBM SPSS Statistics22, accounted for the number of comparisons by multiplying Dunn’s P-value.

## Result

### Video characteristics

**Fig. 2:**
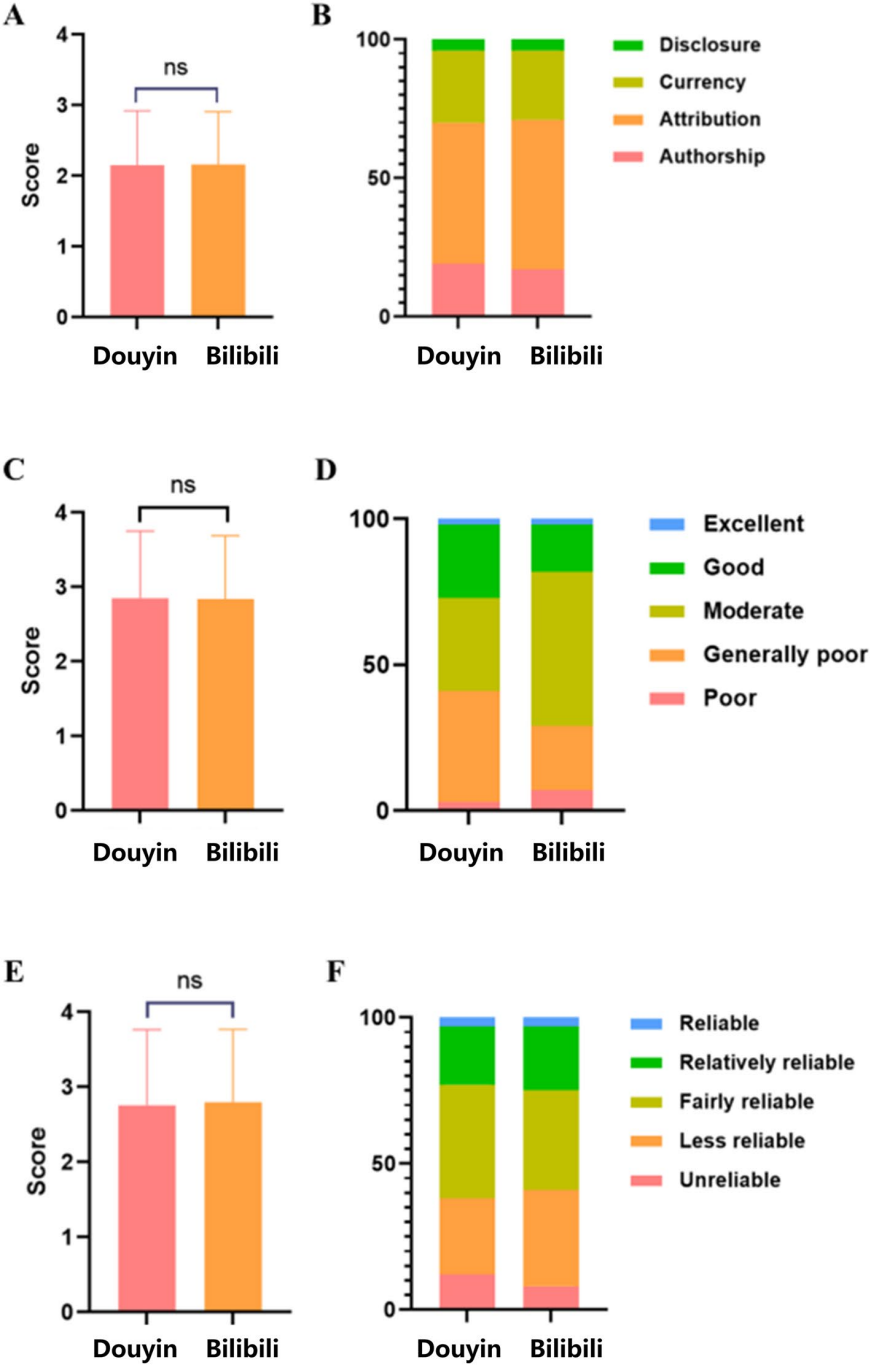
JAMA, GQS, and Modified DISCERN scores of short videos related to lung cancer on Douyin and Bilibili. (**a**) Comparison of JAMA between Douyin and Bilibili videos. (**b**) Proportions of different levels of video quality. (**c**) Comparison of GQS scores between Douyin and Bilibili videos. (**d**) Proportions of different levels of video reliability. (**e**) Comparison of Modified DISCERN scores between Douyin and Bilibili videos. (**f**) Proportions of different levels of video reliability. *P < .05; ns: non-significant.

We sourced a cumulative total of 200 videos pertaining to lung cancer from the platforms Douyin and Bilibili. A comparative analysis of the characteristics of these videos is presented in Table S2. Our findings indicate that videos on Bilibili tend to have an extended duration (*P* < 0.001). Conversely, videos on Douyin surpass those on Bilibili in terms of likes, comments, collections, and shares (*P* < 0.001). Table S3 provides detailed results of the JAMA score, GQS score, and Modified DISCERN score for the videos. Both Douyin and Bilibili videos exhibit identical median scores for JAMA (median: 2.00), GQS (3.00), and Modified DISCERN (3.00). Furthermore, evaluations using JAMA, GQS, and Modified DISCERN did not reveal any statistically significant differences in the information, quality, and reliability of videos between the two platforms (Fig. 2).

### Assessment of video content

**Fig. 3:**
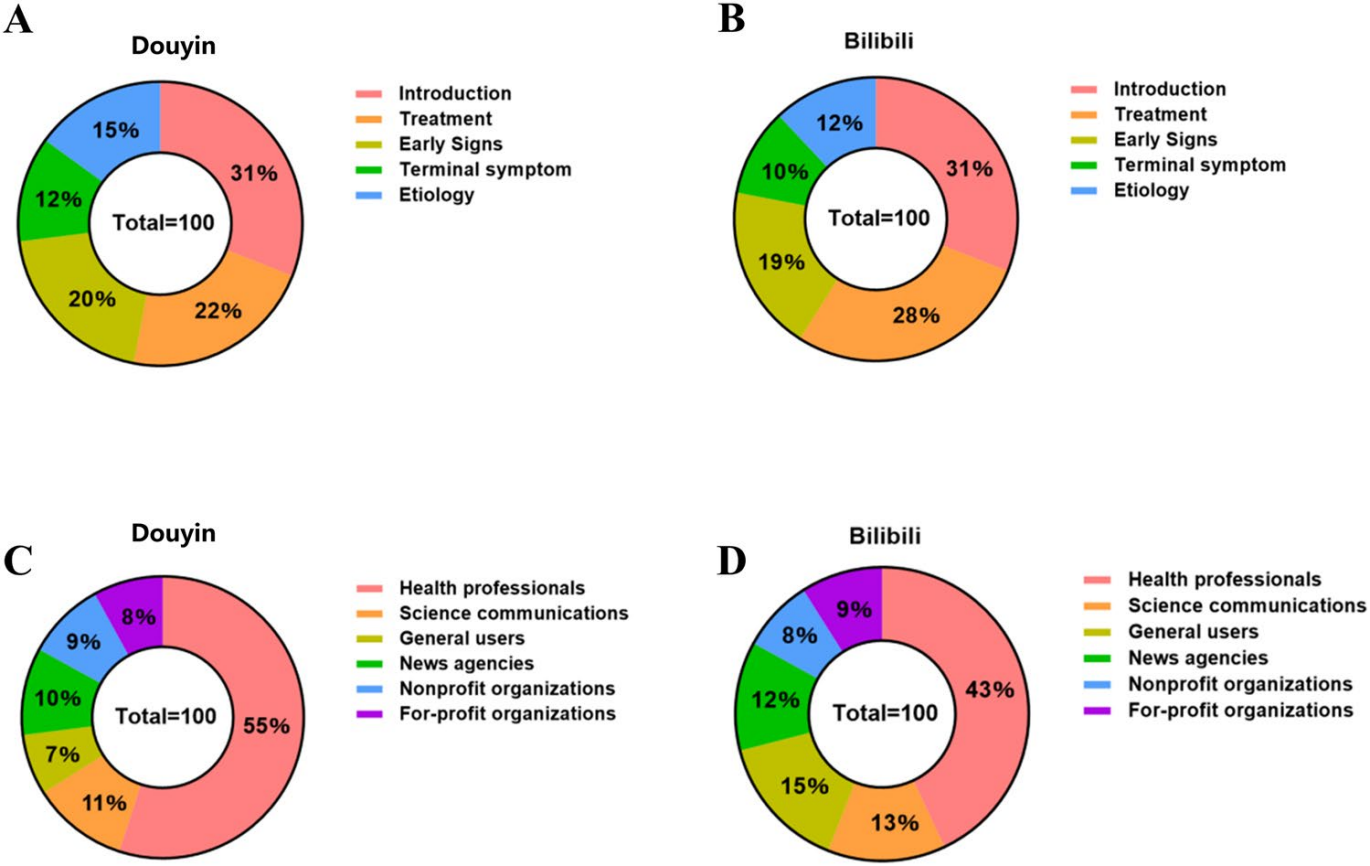
Percentage of videos on lung cancer from different sources and with different contents in Douyin and Bilibili. (**a**) Content types of Douyin videos. (**b**) Content types of Bilibili videos. (**c**) Sources of Douyin videos. (**d**) Sources of Bilibili videos.

In Fig. 3a-b, we compared the fundamental content categories of videos from both Douyin and Bilibili platforms. On Douyin, the majority of the videos were about an introduction to lung cancer (31/100, 31%), followed by treatment (22/100, 22%), early signs (20/100, 20%), etiology (15/100, 15%), and terminal symptom (12/100, 12%). A similar distribution of video content was observed on Bilibili, where most videos were also introduction (31/100, 31%), followed by treatment (28/100, 28%), early signs (19/100, 19%), etiology (12/100, 12%), and terminal symptom (10/100, 10%). The basic data of different video content categories on both platforms are presented in Tables 1 and 2.

**Table 1:** Characteristics of the videos across sources and content in Douyin.

Variable	Days since published	Duration	Likes	Comments	Collections	Shares	JAMA	GQS	Modified DISCERN
**Video content(n = 100), median (range)**									
Introduction(n = 31)	599(1–1176)	71(18–180)	11,000(1879–569,000)	533(50–100,000)	1239(171–57,000)	1431(322–142,000)	2(1–4)	3(1–5)	3(1–5)
Treatment(n = 22)	1060.5(1–1831)	59(31–277)	14,000(1537–1,253,000)	936(100–111,000)	871.5(125–53,000)	2394(74–70,000)	2(1–3)	2.5(1–4)	2.5(1–4)
Early Signs(n = 20)	687(2–1586)	73.5(39–864)	7454(813–937,000)	329(66–57,000)	1091.5(217–144,000)	1091.5(217–144,000)	2(1–3)	3(2–4)	3(2–4)
Terminal symptom(n = 12)	645(472–1174)	114.5(50–342)	35,500(3681–506,000)	2076.5(268–25,000)	1593(113–5360)	2347.5(108–14,000)	1.5(1–3)	2(1–5)	1.5(1–5)
Etiology(n = 15)	717(40–1589)	87(36–278)	89,000(1383–4,614,000)	2165(77–197,000)	2485(92–134,000)	22,000(235–1,694,000)	2(1–4)	3(2–54)	3(1–4)

**Table 2:** Characteristics of the videos across sources and content in Bilibili.

Variable	Days since published	Duration	Likes	Comments	Collections	Shares	JAMA	GQS	Modified DISCERN
**Video content (n = 100), median (range)**									
Introduction(n = 31)	353(52–1102)	207(70–480)	1017(35–41,000)	216(20–6137)	284(12–16,000)	148(16–7262)	2(1–4)	3(1–5)	3(1–5)
Treatment (n = 28)	601(95–1421)	171(44–932)	515(81–12,000)	157.5(23–822)	210(17–3609)	109(3–2447)	2(1–3)	3(1–4)	2(1–4)
Early Signs (n = 19)	634(95–2314)	101(39–349)	390(41–6169)	78(13–709)	200(31–1648)	95(17–481)	2(1–3)	3(2–5)	3(1–5)
Terminal symptom (n = 10)	480.5(15–1320)	204(42–586)	1009.5(156–4086)	171.5(15–1131)	176.5(35–583)	51(14–395)	1.5(1–2)	2(1–4)	2(1–4)
Etiology(n = 12)	695.5(145–1872)	100(53–466)	493.5(52–17,000)	404(12–3581)	364.5(9–2060)	451(18–11,000)	2(2–4)	3(1–4)	3(2–4)

**Fig. 4:**
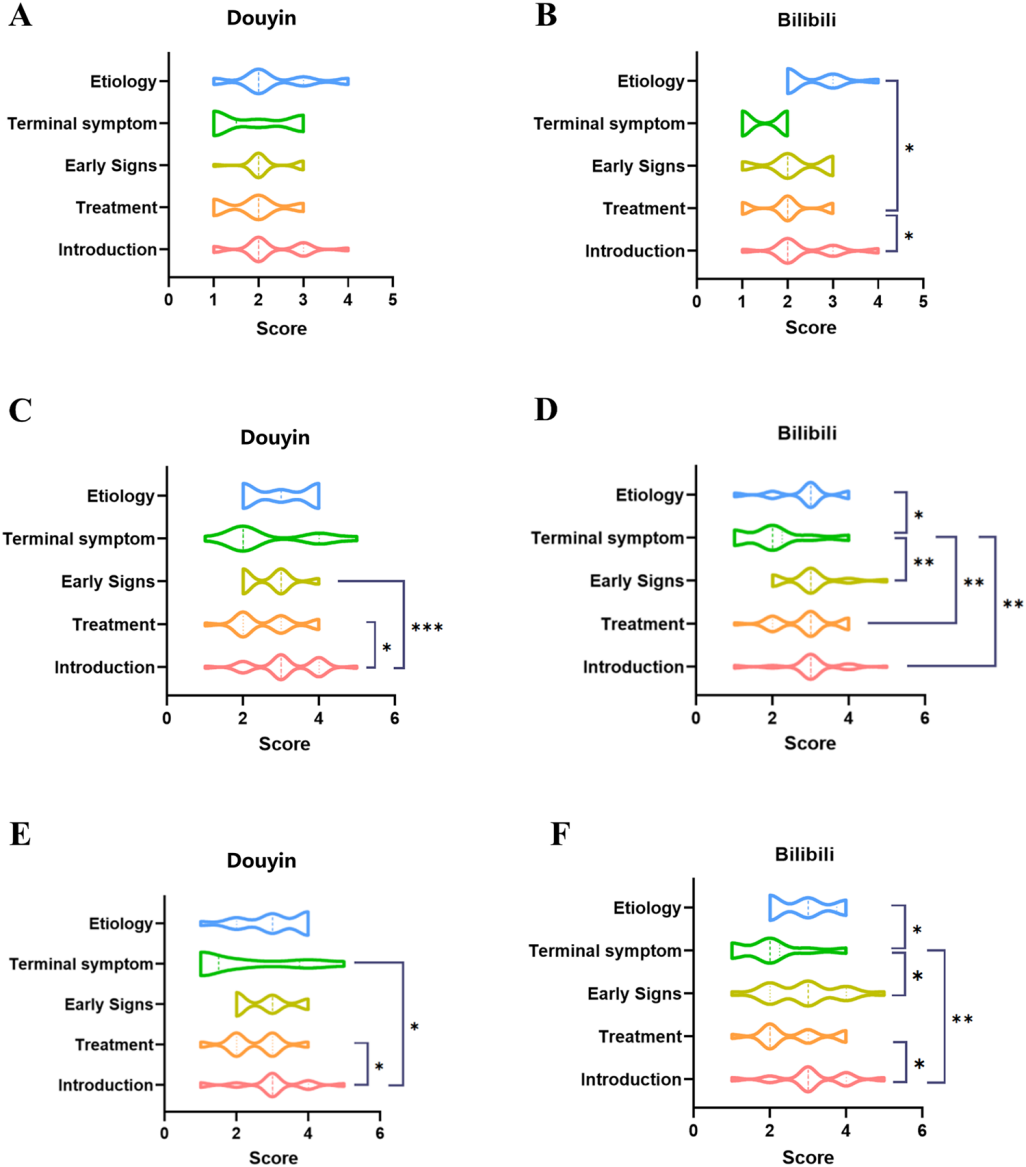
Three evaluation tools’ score of different video content on Douyin and Bilibili. (**a–b**) JAMA score of different video content on Douyin and Bilibili. (**c–d)** GQS score of different video content on Douyin and Bilibili. (**e–f**) Modified DISCERN score of different video content on Douyin and Bilibili. *P < .05, **P < .01, ***P < .001; ns: non-significant.

We conducted an analysis of video content on the two prominent video platforms, On Douyin, videos introducing lung cancer received higher GQS scores compared to those about lung cancer treatment and early signs (*P* < 0.05 and *P* < 0.001, respectively). Moreover, videos about treatment and terminal symptoms obtained higher Modified DISCERN scores than videos about introduction (*P* < 0.05 and *P* < 0.05, respectively) (Fig. 4). Moreover, on Bilibili, content pertaining to etiology and introduction achieved higher JAMA scores compared to treatment videos (*P* < 0.05 and *P* < 0.05, respectively). The GQS scores for videos on terminal symptoms were lower than those for other content categories, indicating poorer video quality for terminal symptom videos (*P* < 0.05). Similarly, videos about terminal symptoms received lower Modified DISCERN scores than videos about etiology, early signs, and introduction (*P* < 0.05, *P* < 0.05, and *P* < 0.01 respectively). Videos on introductions achieved higher Modified DISCERN scores compared to those on treatment (*P* < 0.05) (Fig. 4).

### Analysis of video uploaders

The video uploaders were categorized into six groups: For-profit organizations, Nonprofit organizations, News agencies, General users, Science communications, and Health professionals. We compared the sources of videos on both platforms. On Douyin, videos uploaded by health professionals were the most prevalent (n = 55, 55%), followed by those from science communications (n = 11, 11%) and news agencies (n = 10, 10%). Similarly, on Bilibili, health professionals also uploaded the majority of lung cancer videos (n = 43, 43%), followed by general users (n = 15, 15%) and science communications (n = 13, 13%). Moreover, Bilibili had a higher number of videos from science communications (13 vs. 11), whereas general users tended to produce more videos on Bilibili (15 vs. 7) (Fig. 3c-d).

Table 3 shows that on Douyin, general users contribute videos with the longest average duration, totaling 262 s (*P* = 0.002). Science communications received the most likes (median: 118,000), shares (26,000), and collections (15,000, *P* = 0.001). According to the JAMA scale, For-profit organizations fared best (2.50) and got higher scores than Nonprofit organizations (*P* < 0.05). General users received the lowest ratings, indicating significantly poorer video information compared to other uploaders (*P* = 0.05). Regarding the Modified DISCERN scale, the general users also received lower scores compared to other video uploaders (*P* < 0.05), implying that videos uploaded by general users contain more incomplete information and have lower reliability.

**Table 3:** Comparison of video sources according to video features in Douyin.

Video features (Douyin)	Health professionals	Science communications	General users	News agencies	Nonprofit organizations	For-profit organizations	*P* value
Duration (s, median, range)	70 (28–278)	95 (51–266)	262 (89–864)	50 (25–119)	56 (18–342)	66.5 (39–291)	.002
Likes (median, range)	8666 (813–411,000)	118,000 (11,000–937,000)	58,000 (22,000–1,253,000)	33,500 (2289–4,614,000)	4900 (1313–117,000)	9914.5 (1383–337,000)	.001
Time since video upload day (day, median, range)	717 (1–1498)	615 (100–1374)	484 (428–1011)	792 (143–1831)	600 (499–1182)	1000 (104–1589)	.437
Comments (median, range)	414 (50–32,000)	3188 (254–57,000)	7067 (3094–111,000)	2237 (136–197,000)	488 (66–11,000)	1012 (243–5469)	.001
Shares (median, range)	1257 (74–116,000)	26,000 (2442–218,000)	7807 (1210–26,000)	9629 (871–1,694,000)	1127 (330–3215)	2474 (235–36,000)	.001
Collections (median, range)	676 (92–66,000)	15,000 (752–111,000)	5360 (1699–13,000)	1540 (132–134,000)	683 (152–1494)	1381.5 (289–4849)	.001
JAMA score (median)	2.00	2.00	1.00	2.00	2.00	2.50	.020
GQS score (median)	3.00	3.00	2.00	2.50	3.00	3.00	.121
Modified DISCERN score (median)	3.00	3.00	2.00	2.50	3.00	3.00	.018

**Fig. 5:**
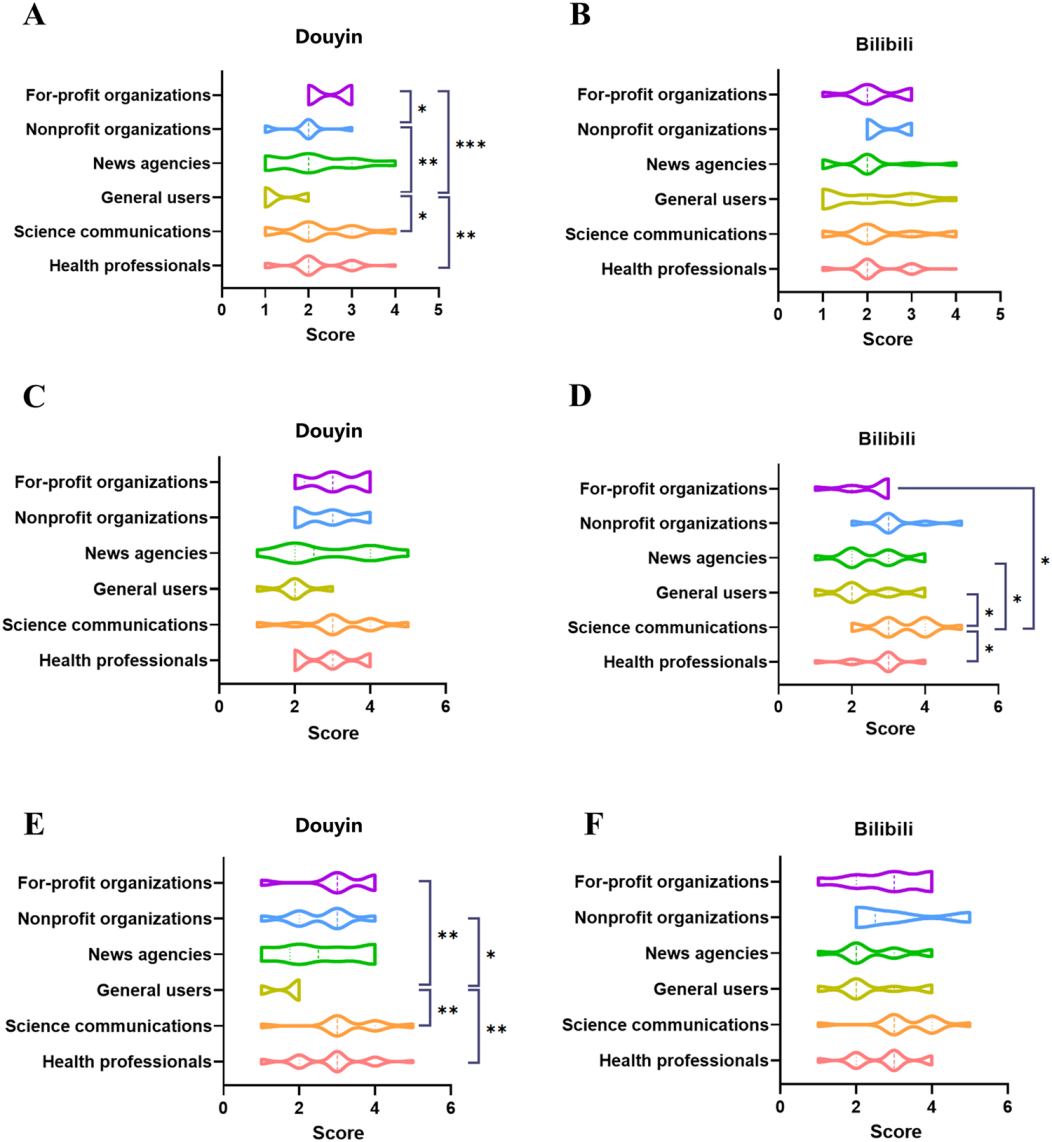
Three evaluation tools’ score of videos related to lung cancer from different video upload sources. (**a–b**) JAMA score of videos from different upload sources. (**c–d**) GQS score of videos from different upload sources. (**e–f**) Modified DISCERN score of videos from different upload sources. *P < .05, **P < .01, ***P < .001; ns: non-significant.

On Bilibili, general users have the longest average video duration (median: 309, *P* = 0.03). News agencies garnered the most collections (4340.5, *P* = 0.003). No statistically significant differences were detected in JAMA scores and Modified DISCERN scores across video uploaders (*P* = 0.850 and *P* = 0.100, respectively). Regarding the GQS scale, videos uploaded by science communications exhibited markedly superior quality compared to those uploaded by general users, health professionals, and news agencies (all *P* < 0.05) (Fig. 5 and Table 4).

**Table 4:** Comparison of video sources according to video features in Bilibili.

Video features (Bilibili)	Health professionals	Science communications	General users	News agencies	Nonprofit organizations	For-profit organizations	*P* value
Duration (s, median, range)	136 (39–819)	114 (70–246)	309 (42–586)	115.5 (81–253)	140.5 (46–306)	220 (64–932)	.030
Likes (median, range)	467 (41–41,000)	575 (35–31,000)	1337 (194–17,000)	4340.5 (127–13,000)	394 (144–7008)	382 (91–11,000)	.003
Time since video upload day (day, median, range)	507 (95–1199)	325 (52–2314)	740 (15–1320)	642.5 (145–1421)	789.5 (101–1872)	606 (80–1972)	.421
Comments (median, range)	89 (12–6137)	172 (23–709)	366 (33–3581)	425.5 (47–2682)	154.5 (13–837)	215 (16–1882)	.050
Shares (median, range)	94 (3–7262)	163 (16–2417)	92 (17–686)	319 (42–11,000)	119 (25–2537)	72 (14–6034)	.176
Collections (median, range)	149 (9–4595)	448 (12–6916)	330 (117–2060)	617.5 (90–1984)	190.5 (45–4707)	147 (31–16,000)	.074
JAMA score (median)	2.00	2.00	2.00	2.00	2.00	2.00	.580
GQS score (median)	3.00	3.00	2.00	2.50	3.00	3.00	.040
Modified DISCERN score (median)	3.00	3.00	2.00	2.00	2.50	3.00	.100

### Correlation analysis

Due to the non-normal distribution of the data, Spearman correlation analysis was utilized to elucidate the relationships among various video variables. Positive correlations were identified among various video variables. Specifically, the duration since video publication was positively correlated with the number of comments (*r* = 0.159, *P* = 0.024) and the number of shares (*r* = 0.195, *P* = 0.006). Notably, a substantial positive correlation was observed between likes and comments (*r* = 0.841, *P* = 0.001). Additionally, strong positive correlations were found between likes and collections (*r* = 0.821, *P* = 0.001) and between likes and shares (*r* = 0.862, *P* = 0.001). Furthermore, there was a notable positive correlation between comments and collections (*r* = 0.738, *P* = 0.001), along with a positive correlation between comments and shares (*r* = 0.783, *P* = 0.001). Likewise, a positive correlation was established between collections and shares (*r* = 0.864, *P* = 0.001). Interestingly, the duration since publication exhibited a negative correlation with video duration (*r* = -0.155, *P* = 0.029), indicating that more recently published videos tended to have shorter durations. Furthermore, video duration showed a negative correlation with the number of shares (*r* = -0.143, *P* = 0.043), suggesting that longer videos had fewer shares (Table 5). Overall, these findings support the notion that engagement metrics such as likes, comments, collections, and shares are interrelated and can influence each other. Additionally, the number of shares demonstrated a positive correlation with JAMA scores (*r* = 0.156, *P* = 0.027). In contrast, the duration since publication exhibited a negative correlation with the GQS scores (*r* = -0.151, *P* = 0.033) (Table 6).

**Table 5:** Spearman correlation analysis between the video variables.

Variable	Days since published	Duration	Likes	Comments	Collections	Shares
Days since published						
*r*	1	− 0.155	0.120	0.159	− 0.053	0.195
*P* value	–	.029	.091	.024	.452	.006
Duration						
*r*	− 0.155	1	− 0.137	0.026	0.053	− 0.143
*P* value	.029	–	.052	.713	.457	.043
Likes						
*r*	0.120	− 0.137	1	0.841	0.841	0.862
*P* value	.091	.052	–	.001	.001	.001
Comments						
*r*	0.159	0.026	0.841	1	0.738	0.783
*P* value	.024	.713	.001	–	.001	.001
Collections						
*r*	0.053	0.053	0.821	0.738	1	0.846
*P* value	.452	.457	.001	.001	–	.001
Shares						
*r*	0.195	− 0.143	0.862	0.783	0.846	1
*P* value	.006	.043	.001	.001	.001	–

**Table 6:** Pearson correlation analysis between video variables and the JAMA, GQS, and Modified DISCERN scores.

Variable	JAMA	GQS	Modified DISCERN
Days since published			
*r*	− 0.055	− 0.151	− 0.065
*P* value	.440	.033	.363
Duration			
*r*	0.044	0.067	0.104
*P* value	.277	.348	.142
Likes			
*r*	0.022	0.010	− 0.008
*P* value	.829	.921	.934
Comments			
*r*	0.037	0.070	0.061
*P* value	.601	.323	.387
Collections			
*r*	0.127	0.128	0.119
*P* value	.072	.071	.092
Shares			
*r*	0.156	0.109	0.103
*P* value	.027	.125	.145

## Discussion

### Principal findings

In this study, we examined the content, reliability, and quality of 200 lung cancer videos sourced from Douyin and Bilibili, two popular short video-sharing platforms in China, using the JAMA, GQS, and Modified DISCERN tools. The findings revealed that the overall quality of lung cancer video content on both platforms was inadequate. This observation may be attributed to the comparatively lenient requirements imposed by the platforms for video release, resulting in insufficient regulation and review of videos. The JAMA, GQS, and Modified DISCERN assessments did not show significant differences in video information, quality, and reliability between the two platforms (JAMA: *P* = 0.933; GQS: *P* = 0.833; Modified DISCERN: *P* = 0.924). However, videos on Bilibili demonstrated lower quality compared to those on Douyin. This discrepancy can be attributed to the higher proportion of health professionals submitting content on Douyin compared to Bilibili, where the majority of users are ordinary consumers rather than professionals. Moreover, despite being shorter in duration, lung cancer videos on Douyin garnered more likes, comments, favorites, and shares compared to those on Bilibili. It is worth noting that Douyin’s platform lacks standardization in verifying the authenticity of users, leading to instances of identity falsification, such as individuals posing as doctors to gain video views while disseminating false information. Although a majority of the lung cancer videos on both Douyin and Bilibili were created by health professionals (Douyin: 55%; Bilibili: 43%), which to some extent ensures the professionalism of the content, further improvements are required to enhance the overall quality and reliability of the videos.

### Quality of the short videos on lung cancer

The findings of this study highlight concerns regarding the overall quality of information presented in videos on Douyin and Bilibili. Our analysis identified several factors contributing to the observed low quality. Videos from health professionals generally exhibited high-quality content, whereas those from other sources demonstrated relatively lower quality, indicating a direct correlation between the expertise of uploaders and video quality. However, we also observed that these platforms did not establish strict threshold requirements for uploaders, with the proportion of non-health professionals even surpassing half. Implementing stricter verification processes for uploaders would enhance the credibility of healthcare information videos and help mitigate the spread of misinformation. Additionally, users of short video platforms often lack the expertise to discern high-quality information and tend to gravitate towards low-quality news reports or patient self-reports. Taking into account the busy schedules of doctors, we propose that public hospitals employ dedicated staff members with expertise in engagingly communicating health knowledge. These professionals would be able to capture the attention of viewers and utilize the influence of social media channels to promote public health more effectively.

### Correlation between video quality and video characteristics

During our data analysis, an interesting observation emerged: more popular videos tend to exhibit lower quality. This finding is consistent with previous research conducted on Douyin^18^. We hypothesize that this phenomenon may be attributed to the fact that educational videos often lack the appeal and interactivity that captivating videos possess, ultimately making it difficult to capture users’ attention. Notably, the recommendation algorithms employed by video-sharing platforms can exacerbate this issue. When certain low-quality videos accumulate a significant number of likes, shares, comments, and other forms of engagement, the platform’s recommendation system is more likely to promote these videos to users, thereby further boosting their popularity and widening the gap between video quality and popularity^19^. Additionally, these video-sharing platforms typically prioritize content related to everyday life and entertainment, as these topics tend to captivate users more effectively. Conversely, educational videos often lack the engaging elements necessary to attract and retain users’ attention, contributing to their relative lack of popularity.

### Evaluation of quantitative scoring tools

In this study, we employed the JAMA, GQS, and Modified DISCERN tools to evaluate the quality and reliability of video content. Our findings indicated that the consistency scores derived from the Modified DISCERN and GQS tools were within acceptable limits. Evaluating the inherent quality of the videos necessitates a manual review conducted by a trained professional. Furthermore, using these tools to objectively evaluate short videos, which often lack comprehensive content, presents specific challenges. Additionally, we observed that the JAMA score, which incorporates four indicators but lacks precision, does not accurately assess the quality of video messages. This observation is consistent with previous research^20^.

### Practical significance

In the dissemination of lung cancer-related short videos, Bilibili and Douyin each have unique advantages. Bilibili provides comprehensive and in-depth medical knowledge through longer videos, enhancing public understanding of the complexity and severity of lung cancer, thereby promoting medical literacy and health awareness. In contrast, Douyin rapidly captures attention with its concise video format and entertaining content, effectively disseminating crucial information to increase public attention and basic awareness of lung cancer, while reaching a broader audience. By combining Bilibili’s detailed content with Douyin’s wide reach, a multi-level health information dissemination strategy can be developed to enhance public awareness of lung cancer prevention and treatment, thus improving overall health education effectiveness.

The development of Internet technology and the increasing demand for health information have elevated the importance of video-sharing platforms as sources of knowledge for patients regarding disease self-management^21^. Videos convey information through visual and auditory means rather than text, facilitating better comprehension and accessibility for patients. Additionally, Health professionals can also utilize these platforms to disseminate professional and scientific knowledge to patients. However, the inconsistency in video quality remains a prevalent issue, with some individuals spreading misinformation to gain views or promote products. To increase the dependability of medical professional videos and guarantee that patients receive scientific and understandable video information, the video review system must be improved, and users who disseminate medical expertise must be properly certified. the inconsistency in video quality remains a prevalent issue, with some individuals spreading misinformation to gain views or promote products. Furthermore, platforms should prioritize properly reviewed videos in search results, thereby promoting the dissemination of credible health knowledge.

### Limitations and future research

The scope of this study is restricted to the two major short video-sharing platforms (Douyin and Bilibili) in China. However, several limitations need to be acknowledged. Firstly, the limited number of videos selected for the study increases the level of uncertainty surrounding its findings. Simultaneously, during the process of including videos, efforts were made to exclude those that did not meet the criteria. However, some videos containing false information may have been inadvertently included, potentially affecting the authenticity of the results. Moreover, video platforms prioritize customized recommendations when displaying content, which warrants reflection and further development in the future. Secondly, three tools—JAMA, GQS, and Modified DISCERN—were selected due to their demonstrated strong performance in prior research evaluating the quality of health-related video material^22^. These methods were used to evaluate the dependability and content quality of the videos. However, these three evaluation tools were initially designed to examine text-based content and therefore have limitations when applied to video content. To mitigate the impact of these limitations, other assessment tools, such as HONcode, could be considered^23^, and the scope of investigation for video assessment could be broadened. Finally, since this research is limited to China and cannot be generalized to lung cancer-related videos in other languages or on other video-sharing platforms. Consequently, further studies specifically targeting other language platforms are essential. The advantage of this study over other tumor-related research lies in its focus on lung cancer, a disease with the highest morbidity and mortality rates, poorer prognosis, and greater relevance. Improving lung cancer patient’s chances of survival and prognosis has significant social and public health implications, given that lung cancer is one of the most prevalent diseases. Therefore, our work is highly significant in improving public health and advancing the prevention and treatment of lung cancer.

Given that current video-sharing platforms are primarily dominated by entertainment content, the number of medical videos is limited. Consequently, expanding the sample size, employing multiple assessment tools, and conducting studies targeting other language platforms are essential. These measures will enhance the comprehensiveness and reliability of future research.

## Conclusion

The objective of this study was to assess the informational quality of 200 lung cancer-related videos posted on Douyin and Bilibili, two of Chinese most popular video-sharing platforms. The results indicated that the informational quality of these videos is generally inadequate. In terms of completeness and content quality, videos from science communications and health professionals were found to be more reliable compared to those from other sources. To increase patient knowledge of lung cancer, doctors and hospitals should actively participate in the growing popularity of video-sharing platforms and offer better-quality videos. Patients seeking healthcare information on these platforms should critically evaluate the scientific validity of the videos and exercise caution. Videos created by health professionals and science communicators are highly recommended over those from other sources.

## Supplementary Information

Supplementary Information 1.

Supplementary Information 2.

Supplementary Information 3.

## Data Availability

The datasets generated and/or analysed during the current study are not publicly available due the confidentiality of the data but are available from the corresponding author on reasonable request.
