# Supplementary material for: Douyin and Bilibili as sources of information on lung cancer in China through assessment and analysis of the content and quality
Source: Sci Rep. 2024 Sep 4;14:20604. doi: 10.1038/s41598-024-70640-y (PMC11375008; doi:10.1038/s41598-024-70640-y)
Supplement: Supplementary file 1 — Supplementary Information 1. [file 41598_2024_70640_MOESM1_ESM.docx]

Table S1: Standard scales used for evaluation of Douyin and Bilibili videos.

| The Journal of the American Medical  Association (JAMA) benchmarks  criteria (1 point for each criterion with  a total score of 4 points.) | Global Quality Scale (GQS) The score ranges from 1 (poor  quality) to 5 (excellent flow and quality) | Modified DISCERN (1 point is given for  every Yes and 0 points for No) |
| --- | --- | --- |
| Authorship: Author and contributor  credentials and their affiliations  should be provided. | Poor quality, poor flow of the site, most information missing,  not at all useful for patients. Score: 1 | Is the video clear, concise, and  understandable? |
| Attribution: Clearly lists all copyright  information and states references  and sources for content. | Generally poor quality and poor flow, some information  listed but many important topics missing, of very limited  use to patients. Score: 2 | Are reliable sources of information  used? (i.e., publication cited,  speaker is specialist) |
| Currency: Initial date of posted content  and subsequent updates to content  should be provided. | Moderate quality, suboptimal flow, some important  information is adequately discussed but others poorly  discussed, somewhat useful for patients. Score: 3 | Is the information presented balanced  and unbiased? |
| Disclosure: Conflicts of interest,  funding, sponsorship, advertising,  support, and video ownership  should be fully disclosed. | Good quality and generally good flow, most of the relevant  information is listed, but some topics not covered, are useful  for patients. Score: 4 | Are additional sources of information  listed for patient reference? |
|  | Excellent quality and excellent flow, very useful for patients.  Score: 5 | Are areas of uncertainty/controversy  mentioned? |
