# Supplementary material for: Douyin and Bilibili as sources of information on lung cancer in China through assessment and analysis of the content and quality
Source: Sci Rep. 2024 Sep 4;14:20604. doi: 10.1038/s41598-024-70640-y (PMC11375008; doi:10.1038/s41598-024-70640-y)
Supplement: Supplementary file 2 — Supplementary Information 2. [file 41598_2024_70640_MOESM2_ESM.docx]

Table S2: Comparison of video sources in two platforms.

| **Video features, median(range)** | **Douyin** | **Bilibili** | ***P* value** |
| --- | --- | --- | --- |
| Duration | 107.40(18-864) | 192.27(39-932) | .001 |
| Likes | 126086.60(813-4614000) | 2745.21(35-41000) | .001 |
| Comments | 7593.02(50-197000) | 413.16(12-6137) | .001 |
| Collections | 6826.22(92-134000) | 797.93(9-16000) | .001 |
| Shares | 31634.68(74-1694000) | 622.54(3-11000) | .001 |
| JAMA score | 2.00(1-4) | 2.00(1-4) | .933 |
| GQS score | 3.00(1-5) | 3.00(1-5) | .833 |
| Modified DISCERN score | 3.00(1-5) | 3.00(1-5) | .924 |
