# Supplementary material for: Douyin and Bilibili as sources of information on lung cancer in China through assessment and analysis of the content and quality
Source: Sci Rep. 2024 Sep 4;14:20604. doi: 10.1038/s41598-024-70640-y (PMC11375008; doi:10.1038/s41598-024-70640-y)
Supplement: Supplementary file 3 — Supplementary Information 3. [file 41598_2024_70640_MOESM3_ESM.docx]

Table S3: The JAMA, GQS, and DISCERN scores for Douyin and Bilibili videos related to lung cancer.

| **Scale, score** | **Douyin(n=100), n** | **Bilibili(n=100), n** |
| --- | --- | --- |
| **JAMA** |  |  |
| 1 | 19 | 17 |
| 2 | 51 | 54 |
| 3 | 26 | 25 |
| 4 | 4 | 4 |
| **GQS** |  |  |
| 1 | 3 | 7 |
| 2 | 38 | 22 |
| 3 | 32 | 53 |
| 4 | 25 | 16 |
| 5 | 2 | 2 |
| **Modified DISCERN** |  |  |
| 1 | 12 | 8 |
| 2 | 26 | 33 |
| 3 | 39 | 34 |
| 4 | 20 | 22 |
| 5 | 3 | 3 |
